# Skeletal Muscle Mass Loss and Physical Function in Young to Middle-Aged Adult Patients With Diabetes: Cross-Sectional Observational Study

**DOI:** 10.2196/58038

**Published:** 2024-12-18

**Authors:** Aki Naruse, Yuka Yamada, Takeshi Miyamoto

**Affiliations:** 1 Division of Medical Technology, Department of Rehabilitation Technology Kumamoto University Hospital Kumamoto Japan; 2 Department of Rehabilitation Medicine Keio University School of Medicine Tokyo Japan; 3 Department of Orthopedic Surgery, Faculty of Life Sciences Kumamoto University Kumamoto Japan

**Keywords:** type 2 diabetes mellitus, middle-aged adults, physical function, skeletal muscle mass, sarcopenia

## Abstract

**Background:**

Recently, it has been reported that older adults with type 2 diabetes mellitus (T2DM) have lower skeletal muscle mass than healthy individuals. Although skeletal muscle mass in older adults with diabetes is occasionally reported, similar reports on young to middle-aged adults are limited.

**Objective:**

This study aims to assess the prevalence of skeletal muscle loss in young to middle-aged adults with diabetes, examine the relationship between skeletal muscle loss and physical function in these patients, and examine whether there are differences in these characteristics between men and women.

**Methods:**

This cross-sectional, observational study included patients younger than 65 years with T2DM who were admitted to our hospital between 2014 and 2022 for educational admission for glycemic control and requested rehabilitation by the Department of Metabolic Medicine. The control group consisted of patients who received rehabilitation during their hospitalization at our hospital and did not have diabetes. The main parameters included skeletal muscle mass, muscle strength, physical function, and activities of daily living.

**Results:**

The prevalence of skeletal muscle mass loss in this study was 18.2% (10/55) in men and 7.7% (4/52) in women. The skeletal muscle mass index (SMI) was 7.7 (SD 0.8) and 8.4 (SD 0.5) for men in the T2DM and control groups, respectively, and 7.0 (SD 0.9) and 6.8 (SD 0.7) for women in the T2DM and control groups, respectively. Therefore, compared with the nondiabetes group, a significant difference was observed in men but not in women (men: *P*<.001, women: *P*=.35). Nonetheless, the diabetes group exhibited significantly lower physical functions, such as a walking speed of 1.3 (SD 0.2) m/s and 1.2 (SD 0.43) m/s for men and women in the T2DM group and 1.6 (SD 0.2) m/s and 1.5 (SD 0.1) m/s for men and women in the control group, respectively (men: *P*<.001, women: *P*<.001). One-leg standing time was measured as 30.7 (SD 26.9) seconds and 29.4 (SD 25.5) seconds for men and women in the T2DM group, compared with 100.5 (SD 30.6) seconds and 82.5 (SD 39.8) seconds for men and women in the control group, respectively, with the T2DM group’s times being significantly lower (men: *P*<.001, women: *P*<.001). Univariate logistic regression analysis showed that SMI was significantly associated with age, BMI, and peripheral neuropathy (all *P*≤.002). Multiple logistic regression analysis showed that BMI exhibited the strongest association (odds ratio 1.15, 95% CI 1.07-1.23; *P*<.001), and peripheral neuropathy was also significantly associated with SMI (*P*=.009).

**Conclusions:**

Patients with diabetes, even those who are not older adults, face an elevated rate of skeletal muscle mass loss, muscle weakness, and a decline in physical function; moreover, they are susceptible to dynapenia and presarcopenia. Therefore, early intervention focusing on muscle evaluation and exercise is crucial.

## Introduction

The global diabetes population is estimated to be 536.6 million, with ~1 (10.5%) in 10 adults having diabetes, according to new estimates published by the International Diabetes Federation [1]. This number is expected to increase to 643 million (11.3%) by 2030 and 783 million (12.2%) by 2045. Furthermore, diabetes is expected to cause 6.7 million deaths in 2021 and is the leading cause of US $966 billion in health care costs during this year [1]. Notably, more than 90% of people with diabetes worldwide have type 2 diabetes mellitus (T2DM) [1], which is characterized by insulin resistance, inflammation, hyperglycemic product accumulation, and increased oxidative stress [2,3]. In particular, insulin resistance causes insulin deficiency, which increases blood glucose levels and leads to overall skeletal muscle weakness [4,5]. Moreover, older adult patients with T2DM have recently been reported to have lesser skeletal muscle mass than healthy controls [6-8]. Besides, skeletal muscle plays an important role in blood glucose metabolism, and reduced skeletal muscle mass has recently been suggested to be a negative predictor of diabetes-related complications [6,7,9]. Specifically, previous studies have reported an association between poor glycemic control [10], renal function [5], and osteoporosis [6]. Additionally, low skeletal muscle mass has been found to be associated with all-cause mortality in patients with T2DM [11]. Therefore, it is important to appropriately treat patients with T2DM, particularly those with reduced skeletal muscle mass. Characterization of patients at a high risk of skeletal muscle mass loss will enable early and aggressive exercise therapy and instruction, which may positively impact life expectancy and related complications [12]. Nevertheless, most previous studies reporting on diabetes and skeletal muscle loss have been conducted in older adult patients with T2DM [7,13-16], few reports have focused on skeletal muscle mass in young to middle-aged adults with T2DM, and skeletal muscle mass loss and related factors have not been clarified. Therefore, the main purpose of this study was to clarify the existence of persons with low skeletal muscle mass in young to middle-aged adult with T2DM, examine the relationship between low skeletal muscle mass and physical function, and examine whether there are differences in these characteristics between men and women. Clarification of these issues could establish the need for early intervention and lead to preventive effects.

## Methods

### Study Design

This study was a single-center, cross-sectional, observational study.

### Setting and Participants

The participants were 107 young to middle-aged adults with T2DM younger than 65 years who were admitted to the Department of Metabolic Medicine, Kumamoto University Hospital, for educational purposes and who requested rehabilitation by the Department of Metabolic Medicine. The study period was from April 2014 to March 2022, and patients were selected retrospectively based on their medical records. The exclusion criteria were patients with preexisting or primary diseases that could affect the skeletal muscle mass index (SMI) by bioelectrical impedance analysis, including patients (1) undergoing cancer treatment, (2) with neurological diseases, (3) with edema, and (4) with internal metal stents or pacemakers implanted in their bodies. The control group consisted of patients who received rehabilitation during their hospitalization at our hospital and did not have diabetes; they were selected at comparable eligible ages, and those who met the above exclusion criteria were excluded. Data for all patients were taken from the first measurement taken after admission.

**Figure 1:**
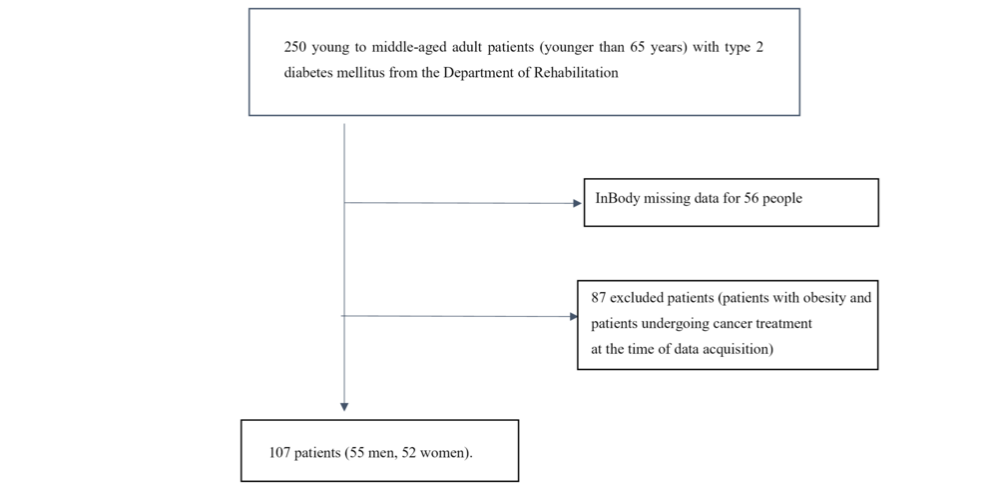
Selection of young to middle-aged adult patients with diabetes.

Of the 250 patients with T2DM who requested rehabilitation by the Department of Metabolic Medicine during the period mentioned above, patients younger than 65 years were included and selected based on the inclusion criteria described above. A total of 56 patients were excluded due to missing data from the muscle mass measurement device, and an additional 87 patients were excluded because they were undergoing cancer treatment, or because of the presence of metals in their bodies, such as pins and screws. Finally, data from 55 men and 52 women were analyzed (Figure 1).

### Variables

This study defines diabetes as exposure and outcome as skeletal muscle mass, which was the main skeletal muscle mass measured using the InBody 720 and InBody S10 (InBody Japan Inc) as the multifrequency bioelectrical impedance analysis method. InBody measurements were performed in the same limb position, using the same procedure, and were performed by a person skilled in operating the equipment. Skeletal muscle mass loss was defined as SMI <7.0 kg/m^2^ in men and <5.7kg/m^2^ in women based on Asian Working Group on Sarcopenia (AWGS) criteria [17]. The assessment items were age, sex, BMI, SMI, physical function (grip strength, comfortable walking speed, and one-leg standing time with eyes opened), medical history, insulin therapy, blood data, pulse wave velocity (PWV), and diabetes-related complications (retinopathy, renal impairment, or neuropathy).

### Measurement

Data on age, sex, BMI, and nutritional status were collected from medical records. BMI was calculated as weight (kg) divided by the square of height (m). Serum albumin and total cholesterol levels were extracted from the blood data as indicators of nutritional status, and estimated glomerular filtration rate levels were extracted from medical records to assess renal function. Blood samples were collected after overnight fasting at admission.

Physical function was assessed based on grip strength, walking speed, and one-leg standing time. Grip strength was measured twice (once on each side), and the best value was used. The 10-m walking speed was measured using a stopwatch, and the test ended when the leading foot stepped on or crossed the 10-m starting line. The test was performed at normal walking speed, measured twice, and the better value was used. One-leg standing time was measured as the time that one leg was raised about 5 cm from the floor, and the better value of the 2 measurements, both left and right, was used. The cutoff values for grip strength and walking speed were also based on those proposed by AWGS as follows: grip strength <26 kg for men and <18 kg for women, and walking speed 0.8 m/s for men and women [17]. In the one-leg standing time, the standard value was 30 seconds or more based on previous studies [18].

To assess glycemic control status, data on hemoglobin A_1c_ (HbA_1c_; glycated hemoglobin), history of diabetes mellitus, and treatment with insulin or oral medications were extracted. History of diabetes was defined as the period from the diagnosis of diabetes mellitus. Furthermore, an ophthalmologist diagnosed diabetic retinopathy as simple retinopathy, preproliferative retinopathy, or proliferative retinopathy. Diabetic nephropathy was graded in severity based on urinary protein or albumin levels and estimated glomerular filtration rate levels and staged from stage I to stage V. Patients were identified at admission. Diabetic peripheral neuropathy was defined as the presence of diabetes mellitus as an essential condition and peripheral neuropathy other than diabetic neuropathy could be ruled out or loss of bilateral Achilles tendon reflexes was defined as having diabetic peripheral neuropathy. Moreover, arteriosclerosis is a complication of diabetes-related vascular diseases, and PWV was extracted to evaluate it. PWV, a test to determine the degree of arterial stiffness, measures the speed at which the heartbeat is transmitted through the arteries to the hands and feet, with higher readings indicating stiffer blood vessels and more advanced arterial stiffness [19].

### Statistical Analysis

All statistical analyses were performed using EZR (Saitama Medical Center, Jichi Medical University), which is a graphical user interface for R (The R Foundation for Statistical Computing). More precisely, it is a modified version of R commander designed to add statistical functions frequently used in biostatistics. The Kolmogorov-Smirnov test was used to assess the normality of the distribution of variables in each group. To test for differences between the groups with and without diabetes, normally distributed data were analyzed using the 2-tailed *t* test, whereas nonnormally distributed data were analyzed using the Mann-Whitney *U* test. Univariate logistic regression analysis was performed to determine the variables associated with SMI. In the multivariate analysis, we set age as a confounding variable and BMI as a mediator. Based on the univariate analysis, clinical findings that were statistically significantly associated with SMI were set as exposures, and the final model was constructed while accounting for multicollinearity. Missing data were excluded at the time of collection and are not included in the statistics. Statistical significance (*P* value) and odds ratios with 95% CI were calculated. In addition, sensitivity analysis was conducted by changing explanatory variables in the multivariate analysis. All results were considered statistically significant at *P*<.05.

### Ethical Considerations

This study was approved by the Ethics Committee of the Graduate School of Life Sciences Research at Kumamoto University (ethics 2366) and complied with the ethical regulations of the Declaration of Helsinki (as revised in Fortaleza, Brazil, October 2013). All survey items and images used in this study were those used in routine medical practice and were conducted so that individuals could not be identified.

As for informed consent, the physician in charge of the medical care concerned conducted the research in accordance with the “Declaration of Helsinki” and the “Ethical Guidelines for Medical Research Involving Human Subjects” based on a separately specified consent document prior to conducting the research. Participation in the study was conducted on an opt-out basis, with a request for cooperation in the clinical research described on the hospital’s website. The open document was published on the website of the Department of Rehabilitation Medicine. The opportunity to refuse participation was guaranteed by stating that if the patient did not wish to cooperate in the research, he or she would be contacted and would not be disadvantaged in any way, with no impact on his or her medical treatment, even if he or she refused to participate in the study. For secondary analyses that use existing data with primary consent, the initial consent covers the secondary analysis without additional consent.

## Results

The characteristics of the T2DM and control groups are shown, stratified by sex (Table 1). The control group comprised 32 (20 men and 12 women) patients without diabetes younger than 65 years who were excluded based on the same criteria. The mean ages were 53.2 (SD 10.2) years and 53.2 (SD 10.1) years among the T2DM group and the control group, respectively. Furthermore, the mean age of the women was 49.5 (SD 10.3) years in T2DM group and 46.8 (SD 12.5) years in the control group, with no significant difference between the groups for either sex (all *P*>.05). Moreover, the SMI was 7.7 (SD 0.8) for the men in the T2DM group, 8.4 (SD 0.5) for men in the control group, 7.0 (SD 0.9) for the women in the T2DM group, and 6.8 (SD 0.7) for women in the control group. Additionally, the BMI was significantly higher in the T2DM group than in the control group for both sexes (*P*=.01 for men, *P*=.001 for women), whereas the SMI was significantly lower in the T2DM group among men but not women (*P*<.001 for men, *P*=.35 for women). Serum albumin levels, an indicator of nutritional status, were significantly lower in the T2DM group than in the control group for both men and women (both *P*<.001). Total cholesterol levels were significantly lower in the T2DM group than in the control group (*P*<.001 for men, *P*=.02 for women). In the T2DM group, the average history of diabetes was 12.2 (SD 10.0) years and 9.6 (SD 7.3) years for men and women, respectively, and the number of men and women with and without insulin use and the severity of each diabetic complication is shown, as well as the value of PWV as an assessment of arterial stiffness (Table 1). The prevalence of skeletal muscle mass loss in this study was 18.2% (10/55) in men and 7.7% (4/52) in women. There were no participants with decreased SMI in the control group.

**Table 1:** Clinical characteristics of the study sample.

Characteristics			Men						Women				
			T2DM^a^ (n=55)		Control (n=20)		*P* value	T2DM (n=52)			Control (n=12)		*P* value
**Age (years), mean (SD)**			53.2 (10.2)		53.2 (10.1)		.96	49.5 (10.3)			46.8 (12.5)		.57
**BMI (kg/m^2^), mean (SD)**			27.1 (4.3)		24.7 (3.4)		.01	30.8 (6.5)			24.7 (4.2)		.001
**Skeletal muscle mass index (kg/m^2^), mean (SD)**			7.7 (0.8)		8.4 (0.5)		<.001	7.0 (0.9)			6.8 (0.7)		.35
**Serum albumin (g/dL), mean (SD)**			3.8 (0.58)		4.4 (0.27)		<.001	3.9 (0.3)			4.4 (0.29)		<.001
**Total cholesterol (mg/dl), mean (SD)**			185.6 (51.9)		211.5 (36.27)		<.001	191.2 (44.5)			226.5 (39.1)		.02
**HbA_1c_^b^(%), mean (SD)**			9.4 (2.07)		5.7 (0.34)		.004	9.3 (1.79)			5.6 (0.43)		<.001
**Diabetes duration (years), mean (SD)**			12.2 (10.0)		—^c^		—	9.6 (7.3)			—		—
**Insulin treatment, n (%)**													
	Yes	19 (35)		—		—		14 (27)		—		—	
**Diabetic nephropathy, n (%)**													
	Stage Ⅰ	35 (67)		—		—		35 (67)		—		—	
	Stage Ⅱ	13 (24)		—		—		15 (29)		—		—	
	Stage Ⅲ	8 (15)		—		—		1 (2)		—		—	
	Stage Ⅳ	2 (4)		—		—		1 (2)		—		—	
	Stage Ⅴ	1 (2)		—		—		0		—		—	
**Diabetic retinopathy, n (%)**													
	Normal	35 (63)		—		—		33 (63)		—		—	
	Nonproliferative diabetic retinopathy	7 (13)		—		—		11 (21)		—		—	
	Preproliferative diabetic retinopathy	2 (4)		—		—		1 (2)		—		—	
	Proliferative diabetic retinopathy	11 (20)		—		—		7 (14)		—		—	
**Diabetic neuropathy, n (%)**													
	Yes	25 (45)		—		—		27 (52)		—		—	
**Arteriosclerosis, mean (SD)**			1600.2 (283.2)		—		—	1480.4 (233.8)			—		—

^a^T2DM: type 2 diabetes mellitus.

^b^HbA_1c_: hemoglobin A_1c_.

^c^Not applicable.

Next, a comparison of physical function assessments between groups is presented, stratified by sex (Table 2). The grip strength was 34.2 (SD 9.8) kg and 22.2 (SD 8.5) kg for men and women in the T2DM group, respectively. Conversely, in the control group, the grip strength for men and women was recorded as 42.0 (SD 8.5) kg and 25.2 (SD 4.6) kg, respectively. Similarly, walking speed was observed to be 1.3 (SD 0.2) m/s and 1.2 (SD 0.43) m/s for men and women in the T2DM group, respectively. In the control group, the values for men and women were 1.6 (SD 0.2) m/s and 1.5 (SD 0.1) m/s, respectively. Furthermore, the time taken to stand on one leg was measured as 30.7 (SD 26.9) seconds and 29.4 (SD 25.5) seconds for men and women in the T2DM group, respectively. In contrast, the control group registered times of 100.5 (SD 30.6) seconds and 82.5 (SD 39.8) seconds for men and women, respectively. Importantly, for both men and women, the BMI was significantly higher in the T2DM group than in the control group (men: *P*=.01, women: *P*=.001), and walking speed and one-leg standing time were significantly lower (all *P*<.001). However, muscle mass and strength were significantly lower for men in the T2DM group than those in the control group (both *P*<.001) but a similar trend was not observed among women in the T2DM group versus the control group (*P*=.35 and *P*=.24, respectively). The diagnostic criteria for sarcopenia by AWGS are a decrease in skeletal muscle mass accompanied by a decrease in muscle strength (decreased grip strength) or physical function (decreased walking speed). The prevalence of sarcopenia in this study based on this criterion was 9.1%(5/55) in men and 3.8%(2/52) in women in the T2DM group.

**Table 2:** Characteristics of physical function in the T2DMa and control groups.

Characteristics	Men				Women		
	T2DM (n=55), mean (SD)	Control (n=20), mean (SD)	*P* value	T2DM (n=52), mean (SD)		Control (n=12), mean (SD)	*P* value
Age (years)	53.2 (10.2)	53.2 (10.1)	.96	49.5 (10.3)		46.8 (12.5)	.57
Grip strength (kg)	34.2 (9.8)	42.0 (8.5)	<.001	22.2 (8.5)		25.2 (4.6)	.24
Gait speed (m/s)	1.3 (0.2)	1.6 (0.2)	<.001	1.2 (0.43)		1.5 (0.1)	<.001
One-leg standing time (seconds)	30.7 (26.9)	100.5 (30.6)	<.001	29.4 (25.5)		82.5 (39.8)	<.001

^a^T2DM: type 2 diabetes mellitus.

To examine differences in characteristics between men and women, factors correlated with skeletal muscle mass were examined separately for men and women (Table 3). In men, there was a negative correlation with age, history of disease, diabetic neuropathy, diabetic retinopathy, insulin use, and PWV, and a positive correlation with BMI. In women, on the other hand, age and diabetic peripheral neuropathy were negatively correlated with age and diabetic neuropathy, and positively correlated with BMI, indicating differences in correlating factors between the sexes.

**Table 3:** Correlation between skeletal muscle mass index and each factor by gender.

Characteristics	Men		Women	
	*P* value	*r*	*P* value	*r*
Age	<.001	–0.35	<.001	–0.49
BMI	<.001	0.62	<.001	0.7
HbA_1c_^a^	.53	0.09	.16	–0.2
Serum albumin	.80	0.04	.76	0.05
Insulin treatment	.01	–0.36	.09	0.51
Diabetic retinopathy	<.01	–0.38	.21	–0.18
Diabetic nephropathy	.94	–0.01	.32	0.14
Diabetic polyneuropathy	.02	–0.32	.05	–0.27
Diabetic duration	.04	–0.25	.08	–0.24
PWV^b^	.03	–0.3	.43	–0.02

^a^HbA_1c_: hemoglobin A_1c_.

^b^PWV: pulse wave velocity.

The relationship between skeletal muscle mass loss (SMI loss) and physical function in the entire T2DM group was analyzed. Univariate logistic regression analysis showed that the SMI was significantly associated with age, BMI, and peripheral neuropathy (all *P*≤.002). In the multiple logistic regression analysis, BMI exhibited the strongest association (odds ratio 1.15, 95% CI 1.07-1.23; *P*<.001). Peripheral neuropathy was also significantly associated with SMI (*P*=.009; Table 4).

**Table 4:** Logistic regression analysis of factors related to the presence of muscle mass loss.

Factors	Univariate analysis				Multivariate analysis		
	OR^a^	95% CI	*P* value	OR		95% CI	*P* value
Age	0.948	0.815-0.980	.002	0.976		0.942-1.01	.18
BMI	1.17	1.090-1.240	<.001	1.15		1.07-1.23	<.001
HbA_1c_^b^	0.99	0.832-1.170	.91	—^c^		—	—
Serum albumin	1.41	0.694-2.920	.35	—		—	—
Insulin treatment	0.508	0.2480-1.030	.06	—		—	—
Diabetic retinopathy	0.696	0.347-1.380	.30	—		—	—
Diabetic nephropathy	2.14	0.083-5.550	.60	—		—	—
Diabetic polyneuropathy	0.315	0.156-0.622	.001	0.391		0.19-0.794	.009
Diabetic duration	0.972	0.937-1.010	.13	—		—	—
PWV^d^	0.999	0.998-1.000	.31	—		—	—

^a^OR: odds ratio.

^b^HbA_1c_: hemoglobin A_1c_.

^c^Not applicable.

^d^PWV: pulse wave velocity.

## Discussion

### Principal Findings

This study is one of the few to focus on skeletal muscle mass loss in young to middle-aged adult patients with diabetes. The results showed that a certain percentage of middle-aged patients with diabetes also had decreased skeletal muscle mass, resulting in decreased muscle strength and physical ability compared with healthy individuals. Furthermore, middle-aged men with diabetes had higher rates of skeletal muscle mass loss, muscle weakness, and loss of physical ability than women with diabetes.

According to the AWGS 2019, sarcopenia is defined as a loss of skeletal muscle mass and a loss of muscle strength or physical capacity, while severe sarcopenia is defined as a loss of skeletal muscle mass plus a loss of both muscle strength and physical capacity; moreover, severe sarcopenia is defined as a loss of both muscle strength and physical performance in addition to a loss of skeletal muscle mass. The results of this study suggest that middle-aged patients with diabetes, especially men, have a higher risk for loss of muscle mass and decline in both muscle strength and physical function than those without diabetes. To elucidate the relationship between diabetes and sarcopenia, skeletal muscle mass plays a substantial role in peripheral blood glucose uptake and use, and its reduction in sarcopenia results in compromised glucose tolerance due to diminished peripheral blood glucose uptake. Similarly, reduced physical activity stemming from loss of muscle mass also contributes to impaired glucose tolerance, exacerbating diabetes and leading to difficulty in controlling T2DM. Thus, T2DM promotes the development of sarcopenia, which contributes to the onset and worsening of diabetes, coupled with diabetic complications, leading to a vicious cycle of decreased physical activity, a secondary decrease in skeletal muscle mass, and worsening diabetes [20].

### Comparison With Prior Work

Previous studies have reported that older adult patients with diabetes are more prone to age-related loss of skeletal muscle mass than healthy individuals and that sarcopenia occurs several years earlier in patients with diabetes than in healthy individuals [21]. Furthermore, studies in mice have shown that hyperglycemia, a central disease of diabetes, promotes muscle atrophy via the WWP1/KLF15 pathway and that in mice lacking KLF15, specifically in the skeletal muscle, hyperglycemia-induced muscle atrophy is suppressed [4]. Moreover, it has been reported that terminal glycation products, a major cause of diabetic vascular complications, accumulate with chronic hyperglycemia and aging in various tissues; additionally, their accumulation contributes to a decline in muscle mass and strength, possibly leading to sarcopenia and dynapenia in T2DM [22]. Similar to previous studies on older adult patients with diabetes, this study found that skeletal muscle mass and strength loss occur earlier in young to middle-aged adult patients with diabetes, especially in men, than in healthy individuals. In addition to age, male sex, low BMI, elevated HbA_1c_, diabetic nephropathy, and low serum albumin concentration have been reported as risk factors associated with low muscle strength in patients with diabetes [6]. In contrast, other studies have reported that diabetic polyneuropathy contributes to lower limb muscle weakness in older patients with T2DM [19]. In this study, logistic regression analysis showed that BMI and peripheral neuropathy were associated with skeletal muscle mass loss in middle-aged adult patients with T2DM, similar to the results in older adult patients with T2DM [23]; however, there was no association with HbA_1c_ or diabetic complications. Furthermore, Korean older adults with T2DM had a prevalence of skeletal muscle loss of 57.6% in men and 7.1% in women. The association between T2DM and skeletal muscle loss appeared more prominent in men, with the risk of muscle loss being approximately 2 to 4 times higher in older adults with T2DM than in healthy controls [24]. In this study, the prevalence of skeletal muscle loss in young to middle-aged individuals with T2DM was 18.2% in men and 7.7% in women, suggesting that even in younger populations, T2DM is associated with an increased risk of skeletal muscle mass loss, particularly in men.

This study showed no differences in skeletal muscle mass or muscle strength between middle-aged adult women with T2DM and healthy controls. This outcome was anticipated owing to the younger average age and elevated BMI of the women participants in this study. Moreover, the limited number of control participants used for comparison is also believed to have influenced this result. Nonetheless, despite preserving skeletal muscle mass and strength, the participants notably underperformed, compared with healthy controls, in terms of physical performance and balance function. This indicates that the quality of skeletal muscle may have been compromised. Therefore, the prospect of dynapenia occurring in women was anticipated. Although many previous studies have compared sarcopenia-related factors, such as skeletal muscle mass, muscle strength, and walking speed, few have examined balance function, which makes this study significant. Furthermore, we found that the same functional decline in both men and women differed between those with and without loss of skeletal muscle mass and strength.

### Limitations

This study had some limitations. First, owing to the nature of the study, the number of participants was inadequate, especially in the female control group. This was due to the fact that this was a single-center, backward-looking study, which limited the selection of participants. Moreover, because this was a retrospective study, there were many missing data points, which made it difficult to select the participants.

Finally, this study aimed to elucidate the presence of skeletal muscle mass and muscle weakness in young and middle-aged patients with diabetes. The study also sought to identify associated factors that will be applied in clinical education and exercise therapy for patients with diabetes. Importantly, this information should be considered from an early stage for the purpose of preventive rehabilitation. Therefore, this study shows that young to middle-aged adult patients with diabetes exhibit reduced skeletal muscle mass, muscle weakness, sarcopenia, and loss of physical performance, proving the importance of exercise therapy and the need for strength training. We believe this information can be applied to patient education in future clinical practice. Moreover, as a future prospect, we would like to develop this study further to examine sex differences and develop better teaching and more effective strength training methods.

### Conclusions

In conclusion, young to middle-aged adult patients with diabetes, especially men, have lower skeletal muscle mass than those without diabetes and may experience further decline in physical function. Therefore, early and detailed assessment of muscle mass and strength as well as physical function will be necessary even in young and middle-aged individuals.

## Abbreviations

AWGS: Asian Working Group on Sarcopenia
HbA1c: hemoglobin A1c
PWV: pulse wave velocity
SMI: skeletal muscle mass index
T2DM: type 2 diabetes mellitus

## Data Availability

Data sharing is not applicable to this article as no data sets were generated or analyzed during this study.
